# Active Hydrogen Bond Network (AHBN) and Applications for Improvement of Thermal Stability and pH-Sensitivity of Pullulanase from *Bacillus naganoensis*

**DOI:** 10.1371/journal.pone.0169080

**Published:** 2017-01-19

**Authors:** Qing-Yan Wang, Neng-Zhong Xie, Qi-Shi Du, Yan Qin, Jian-Xiu Li, Jian-Zong Meng, Ri-Bo Huang

**Affiliations:** 1 State Key Laboratory of Biomass Enzyme Technology, National Engineering Research Center for Non-Food Biorefinery, Guangxi Academy of Sciences, Nanning, Guangxi, China; 2 Gordon Life Science Institute, Belmont, MA, United States of America; 3 Life Science and Technology College, Guangxi University, Nanning, Guangxi, China; Russian Academy of Medical Sciences, RUSSIAN FEDERATION

## Abstract

A method, so called “active hydrogen bond network” (AHBN), is proposed for site-directed mutations of hydrolytic enzymes. In an enzyme the AHBN consists of the active residues, functional residues, and conservative water molecules, which are connected by hydrogen bonds, forming a three dimensional network. In the catalysis hydrolytic reactions of hydrolytic enzymes AHBN is responsible for the transportation of protons and water molecules, and maintaining the active and dynamic structures of enzymes. The AHBN of pullulanase BNPulA324 from *Bacillus naganoensis* was constructed based on a homologous model structure using Swiss Model Protein-modeling Server according to the template structure of pullulanase BAPulA (2WAN). The pullulanase BNPulA324 are mutated at the mutation sites selected by means of the AHBN method. Both thermal stability and pH-sensitivity of pullulanase BNPulA324 were successfully improved. The mutations at the residues located at the out edge of AHBN may yield positive effects. On the other hand the mutations at the residues inside the AHBN may deprive the bioactivity of enzymes. The AHBN method, proposed in this study, may provide an assistant and alternate tool for protein rational design and protein engineering.

## Introduction

Cassava [1] is the third largest crop for food starch production, next only to corn and rice. Particularly cassava is one of the most drought-tolerant and high-yield crops, capable of growing on marginal soils, providing the major food starch in many countries in southern America, Africa, and south and south-east Asia [2,3]. In recent years more and more cassava starch is used as the feedstock for biofuel (ethanol and butanol) industry in China [4,5], which is a reasonable solution for the serious problem of air pollution in many Chinese cities. However, cassava starch is rich in branch polymer, amylopectin, one of the largest molecules in nature with an average DP (degree of polymerization) of about 2 million. The molecular weight of amylopectin is about 1000 times as high as the molecular weight of amylose [6]. In addition to α-1,4 bonds, which are present in amylose and the linear segments of amylopectin, amylopectin molecule has α-1,6 bonds, which occur every 20 to 30 anhydroglucose units [7,8].

In ethanol industry usually α-amylase is only capable of catalyzing the hydrolysis of α-1,4 bonds, and pullulanase has to be used to catalyze the hydrolysis of α-1,6 bonds for higher production of amylodextrin. Pullulanase is a specific kind of debranching enzyme, an amylolytic exoenzyme [9–11]. It is known that pullulanase not only possesses activities against pullulan, but also hydrolysis activities against α-1,6-glycosidic linkage in starch, glycogen, amylopectin, as well as against branched oligosaccharides produced by their partial decomposition [8]. Because of this characteristic, pullulanase is called a “debranching enzyme”. Pullulanase is also used as a processing aid in grain processing biotechnology (production of sweeteners and other food products).

Currently, in industry the saccharification reaction is usually carried out at ~60°C and pH ~4.5 over 48 to 60 hours to maximize the activity of the glucoamylase. Thus, a pullulanase with sufficient thermal stability (temperature up to 60–65°C) and broad pH-stability (pH in the range 4.0–5.0), the conditions under which glucoamylase exhibits its maximum activity, is of special interest [12–14]. Improvements of the thermal stability and pH-stability of current pullulanases is one of the central targets in biofuel industry using the cassava starch as the raw materials [15,16]. One way to obtain enzymes with desired characteristics is to reconstruct existing enzymes by protein engineering, which has been successfully utilized to enhance the stability or catalytic efficiency of many enzymes [17–22]. In the hydrolysis catalysis reaction by pullulanases and α-amylases, water molecules and protons are important reactants, which are transported by the hydrogen bond network in the active region of enzymes [23]. In this study a new method, so called “active hydrogen bond network” (AHBN) is proposed for protein engineering. The AHBN method was applied in the engineering of pllulanase BNPulA324 from *Bacillus naganoensis*.

## Materials and Methods

Pullulanase BNPulA324 is prepared from *B*. *naganoensis*, then it is engineered by means of site-directed mutations [24–26] using AHBN method for better thermal stability and pH-stability.

### Bacterial strains, plasmids, and enzymes

Luria-Bertani (LB) media for bacterial culture were purchased from Difco Laboratories (Detroit, MI, USA). The oligonucleotide primers were synthesized by Shanghai Generay Biotech Co., Ltd. (Shanghai, China). *E*. *coli* JM109 was the host for cloning work, and *E*. *coli* BL21(DE3) was the host for the expression of the pullulanase. Plasmid pET-22b(+) was used for subcloning. The Restriction enzymes, DNA polymerase and ligases were purchased fromTaKaRa.

### Gene cloning and construction

To obtain partial pulA gene, PCR was carried out with Primstar DNA polymerase. Pullulanase BNPulA324 (deleted 324 bp encoding the N-terminal 108 amino-acid residues of PulA) was amplified using primers PulA324F and PulA324-R (listed in Table 1). The genomic DNA from *B*. *naganoensis* ATCC53909 was prepared and used as the template. The amplified DNA fragment was digested with *EcoR* I/ *Xho*I and cloned into pET-22b (+) via *EcoR* I and *Xho*I site, which yielded the recombinant plasmids pET22b- *pul*A324.

**Table 1:** Primers for PCR.

Primers	Description
PulA324F	5’-GTAGAATTCACCTGCTGTAAGTAACGC-3’(*EcoR* I)
PulA324R	5’-GTACTCGAGTTTACCATCAGATGGGCT-3’(*Xho*I)
R509X	5’-TACCATATCGACGGCTTCNNKTTCGATCTT ATGGCT-3’
	5’-AACAGCCATAAGATCGAAMNNGAAGCCGTCGATATG-3’
N681X	5’-GGCGGCAACGACNNKAGCTATAATGCTGGTGAT-3’
	5’-AGCATTATAGCTMNNGTCGTTGCCGCCTTTCGT-3’
D570X	5’-GCTGTATTTAATNNKAATCTGCGAAACGGTTTG-3’
	5’-GTTTCGCAGATTMNNATTAAATACAGCCACCTC-3’

Note: X represents one of 19 kinds of amino acids, N represents of the four nucleotides mixed with equal proportion, M and K represent the two nucleotides (A/C and G/T) mixed with equal proportion, separately.

### Design and construction of site-directed mutants

The scheme of site-directed mutations for engineering the pullulanase BNPulA324 from *B*. *naganoensis* ATCC53909 was constructed by means of AHBN method based on a homology model structure, which will be described in detailed in Result section The mutations were generated by QuikChange mutagenesis protocol (Stratagene, La Jolla, CA, USA) and were verified by DNA sequencing. The sequences of oligonucleotides containing the appropriate base changes are listed in Table 1. Plasmid pET22b- *pul*A324 was used as the template.

### Expression and purification

*E*.*coli* BL21(DE3) harboring recombinant plasmid was cultured in LB medium containing 100 μg/ml ampicillin at 37°C until the *A*600 reached 0.6. Protein expression was induced at 30°C with the addition of isopropyl β-D-thiogalactopyranoside (IPTG) to a final concentration of 1.0 mM for 16 h. The culture supernatants were collected by centrifugation and were concentrated with ultrafiltration device (Millipore, US), thus the crude enzymes were obtained. The crude enzyme was then subjected to immobilized metal affinity chromatography (IMAC) utilizing the carboxy terminal His-tag. The IMAC column, containing a 2-ml bed volume of chelating Sepharose (fast flow, GE Healthcare, UK; loaded by equilibrating with NiCl followed by washing with water) per liter of original bacterial culture, was equilibrated with PBS containing 10 mM imidazole and 1 M NaCl (pH 7.5). The crude enzyme adjusted to the same buffer composition and applied to the column. After subsequent washing with ten bed volumes of PBS containing 10 mM imidazole, 1 M NaCl (pH 7.5), ten bed volumes of PBS containing 50 mM imidazole, 1 M NaCl (pH 7.5) and a final washing step with ten bed volumes of PBS, the proteins were eluted from the column using an imidazole concentration of 250 mM in PBS containing 1 M NaCl (pH 7.5) over ten bed volumes and with another ten bed volumes of PBS containing 250 mM imidazole and 1 M NaCl (pH 7.5). The fractions containing pullulanase activity were pooled and dialyzed against buffer A (20 mM phosphate-citric acid buffer, pH 4.8) overnight.

### Pullulanase activity assay

Pullulanase activity was measured in 50 mM phosphate-citric acid buffer (pH 4.8) according to Miller using 1% (wt/vol) pullulan as the substrate at 60°C for 15 min. One unit of pullulanase activity was defined as the amount of enzyme that released 1μM D-glucose reducing sugar equivalents per minute from pullulan, under the defined assay conditions. All parameters presented in this study are the mean values derived from triplicate measurements.

### Effects of pH and temperature on enzyme activity

The optimum pH of the recombinant enzyme was determined by measuring its activity across a pH range of 3.6 to 6.0 (at an interval of 0.2 unit) using sodium phosphate-citric acid buffer. The residual pullulanase activity was measured after the enzyme was incubated in phosphate-citric acid buffer with different pH value, which was used to assess the pH stability. Samples diluted in 50 mM phosphate-citric acid buffer of different pH value (pH 3.5–7.0, at an interval of 0.5 unit) to a protein concentration of 0.05 mg/ml were incubated at 60°C for 5 hours. After buffer treatment, the residual activity for 1.0% pullulan was measured at 60°C in the same buffer (pH 4.8). The initial activity before buffer treatment at 60°C was taken as 100%.

The temperature dependence of the recombinant enzyme activity was measured between 37 and 68°C in 50 mM phosphate-citric acid buffer at pH 4.8 using pullulan as the substrate. At each temperature, the buffer and pullulan were preincubated for 10 min. The reaction was initiated by the addition of the enzyme and allowed to proceed for 15 min. Samples diluted in 50 mM sodium acetate buffer (pH 4.8) to a protein concentration of 0.05 mg/ml were incubated at 60°C for a period of time. After heat treatment, the residual activity for 1.0% pullulan was measured at 60°C in the same buffer, which was used to assess thermostability. The initial activity before heat treatment was taken as 100%. All of the values presented in graphs are the means of three replications.

## Results

**Fig 1:**
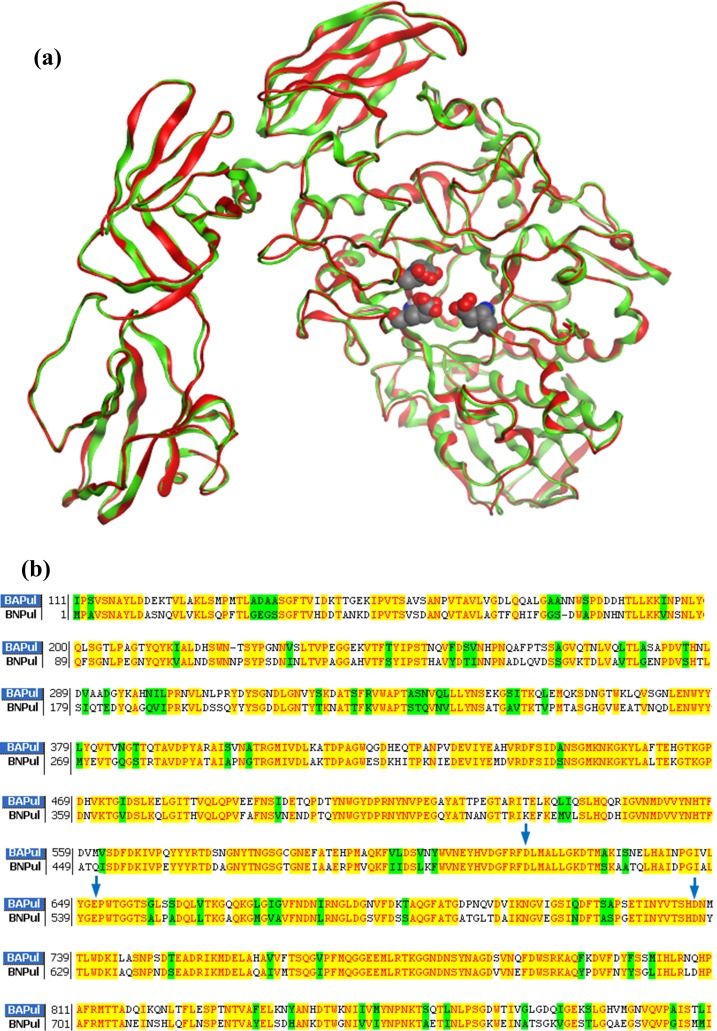
The homology 3D structure of pullulanase BNPulA324 from *bacillus naganoensis*. (a) The homologous structure of pullulanase BNPulA324 was constructed based on the experimental structure of pullulanase BAPulA (2WAN). The three amio acids in ball render are catalysis active residues (Asp511, Glu540, and Asp625). The two peptide backbones of model pullulanase (red) and template pullulanase (green) are consistent very well. (b) The sequence alignment of model pullulanase BNPulA324 and template pullulanase BAPulA (2WAN). The two pullulanases share 65.93% identity.

For convenience in this study all residues are numbered based on the homology structure of pullulanase BNPulA324 whose pdb data are stored in S1 File. The superimposition structural pdb data of model pullulanase BNPulA324 and template pullulanase 2WAN with water molecules are stored in S2 File. The structure and sequence alignments of the model structure and template structure are shown in Fig 1(A) and 1(B), respectively.

### Homology model of pullulanase BNPulA324

Pullulanase BNPulA324 from *B*. *naganoensis* ATCC53909 shares 65.93% homology identity with pullulanase BAPulA (pdb code: 2WAN) [27], which is the highest homology among the experiment structures of pullulanases in NCBI protein data bank (https://www.ncbi.nlm.nih.gov/). Therefore pullulanase 2WAN was used as the template for homology structural construction using software SwissModel Protein-modeling Server (http://swissmodel.expasy.org/). The 3D structural alignment of model and template pullulanases are shown in Fig 1(A), and the sequence alignment of two pullulannases are shown in Fig 1(B). In Fig 1 the two structures of model and template pullulanases are consistent very well, indicating the homology model structure of pullulanase BNPulA324 is reliable. After the homology model [S1 File] was built, the orientations of all residue side chains in model pullulanase were adjusted using energy minimization calculations. The coordinates of water molecules were directly taken from the pdb data of 2WAN. In the model structure the orientations of water molecules were also adjusted using energy minimization calculations.

### Active hydrogen bond network (AHBN) in pullulanase

**Fig 2:**
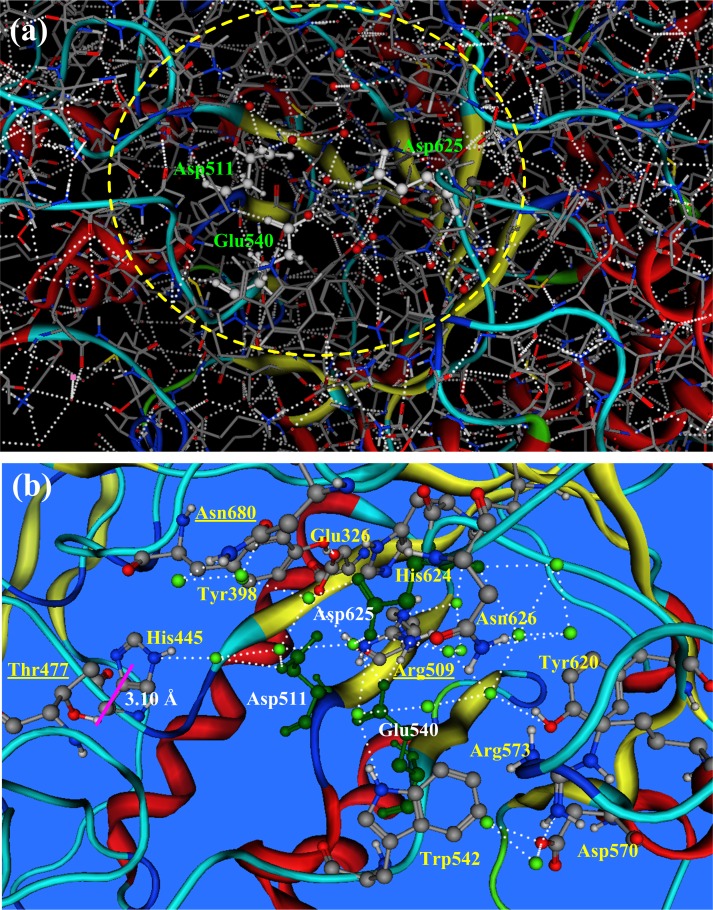
The hydrogrn bond network in pullulanase BNPulA324. (a) The complete hydrogrn bond network in pullulanase BNPulA324. The dashed white lines are the hydrogen bonds that connect the electronegative atoms (oxygen and nitrogen) and polar hydrogen atoms among amino acid residues (side chains and backbones) and water molecules, forming a complicated hydrogen bond network. (b) The active hydrogen bond network (AHBN) in pullulanase BNPulA324.AHBN is the local hydrogen bond network in the active region of protein. The AHBN of pullulanase BNPulA324 consists of three active residues (Asp511, Glu540, and Asp625), twelve functional residues (Glu326, Tyr398, His445, Thr477, Arg509, Trp542, Asp570, Arg573, Tyr620, His624, Asn626, Asn680), and eighteen water molecules (W311, W602, W603, W604, W605, W630, W659, W662, W724, W727, W729, W730, W731, W732, W734, W786, W788, W952).

The hydrogen bond network in a protein can be found using a biological software, e.g., SYBYL-X 2.1 (http://www.tripos.com/). The complete hydrogen bond network in pullulanase BNPulA324 (including crystal water molecules) is shown in Fig 2(A). In Fig 2(A) the dashed white lines are the hydrogen bonds that connect the electronegative atoms (oxygen and nitrogen) and the polar hydrogen atoms in amino acid residues (side chains and backbones) and water molecules. The complete hydrogen bond network in protein is very complicated. However, the hydrogen bond network in the active region of an enzyme is relatively simple and independent. In pullulanases the active region is the place surrounding the three active residues Asp511, Glu540, and Asp625, so called catalysis triad, at the top of (α/β)_8_ TIM barrel. Starting from the three active residues (Asp511, Glu540, and Asp625), tracing the hydrogen bonds, the active hydrogen bond network (AHBN) of pullulanase BNPulA324 is identified, as shown in Fig 2(B). In AHBN the three active residues (Asp511, Glu540, and Asp625), twelve functional residues (Glu326, Tyr398, His445, Thr477, Arg509, Trp542, Asp570, Arg573, Tyr620, His624, Asn626, Asn680), and eighteen water molecules (W311, W602, W603, W604, W605, W630, W659, W662, W724, W727, W729, W730, W731, W732, W734, W786, W788, W952) are cross-linked by the hydrogen bonds. In AHBN each water molecule is connected by 2 to 4 hydrogen bonds. In the hydrolysis reactions of pullulanase AHBN is responsible for the transportation of protons and water molecules, which may influence on the reaction activity, pH-sensitivity and thermal stability of pullulanases.

In the active hydrogen bond network (AHBN) all residues and water molecules are connected by the hydrogen bonds. However, Thr477 is the only exception that is not connected by common hydrogen bond, but by the polar hydrogen-π interaction [28] between the imidazole ring of His445 and the hydroxyl group of Thr477. Among the twelve functional residues, involved in the AHBN, some residues (e.g., Glu326 and Arg509) are located inside the TIM barrel [29–31], while other residues are at the outside of TIM barrel. We pay high attention on the residues at the out edge of AHBN, which may play important roles for the transportation of proton and water from outside to inside of the active region of pullunases. In this study three residues (Thr477, Arg509, and Asn680) are selected from the AHBN for site-directed mutations [24–26] to improve the pH-sensitivity and thermal stability of pullulanase BNPulA324. In the three mutation targeting residues, Arg509 is in the middle of the fourth β-strand of the (α/β)_8_ TIM barrel [32,33]. Other two mutation targeting residues Asn680 and Thr477 are located at the out edge of AHBN.

### Effects of Arg509-mutations on enzyme activity

**Fig 3:**
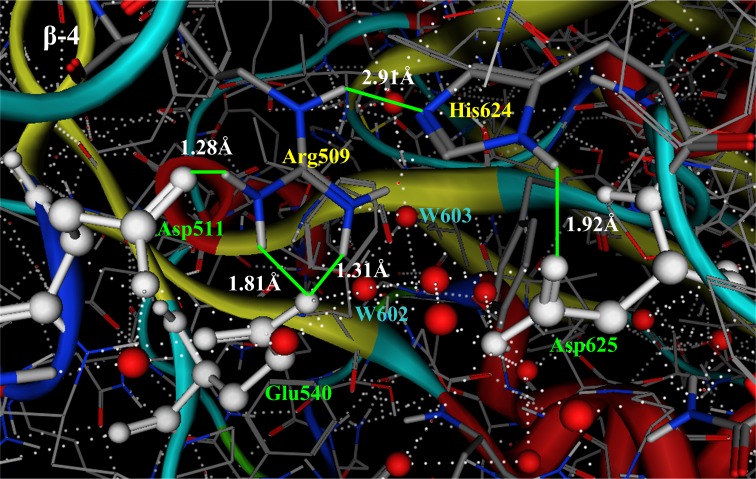
Functional residue Arg509 in AHBN of pullulanase BNPulA324. Arg509 is in the middle of the fourth β-strand of TIM barrel, which directly connects the active residues Asp511 and Glu 540, two water molecules (W602 and W603), and indirectly connects the active residue Asp625 through functional residue His624. Arg509 plays important role in both hydrolysis catalysis reaction and transportation of protons and water molecules.

Arg509 is an important member in the AHBN. As shown in Fig 3, Arg509 is located in the middle of the fourth β-strand in the TIM barrel, which directly connects the active residues Asp511 and Glu540, two water molecules (W602 and W603), and indirectly connects the active residue Asp625 through functional residue His624. Arg509 plays important role in both hydrolysis catalysis reaction and transportation of protons and water molecules. Saturated mutation experiments reveal that any mutation on this position either expropriates bioactivity of pullulanase completely, or minimizes its bioactivity seriously.

### Effects of Asn680-mutations on enzyme activity

**Fig 4:**
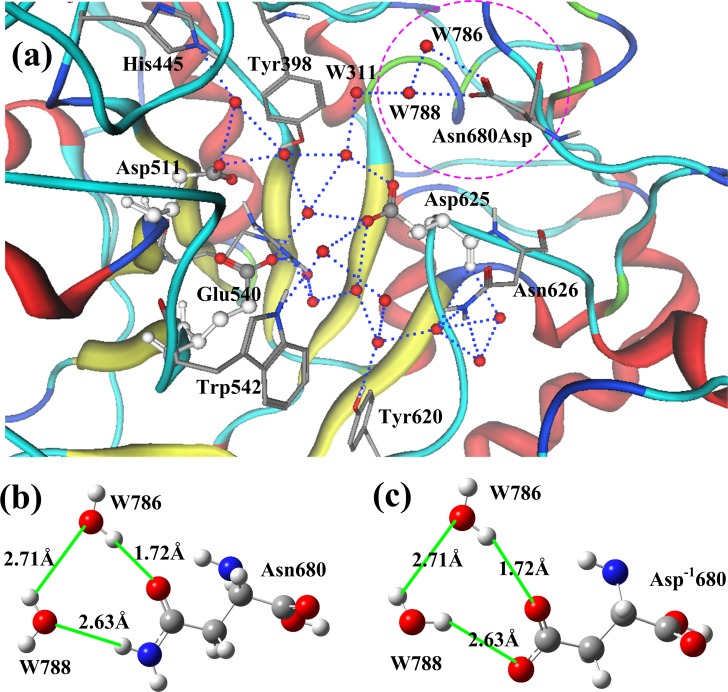
Functional residue Asn680 in AHBN of pullulanase BNPulA324. (a) Position of Asn680 (Asp680) in AHBN. Asn680 is located at the out edge of the AHBN, connecting two water molecules W786 and W788. Asn680 has no directive influence on the active residues. However it may affect the bioactivity of pullulanase through the AHBN. (b) Interaction structure between water molecules (W786 and W788) and Asn680. (c) Interaction structure between water molecules (W786 and W788) and Asp680. Based on the energy minimization calculations, mutation Asn680Asp changes the orientation and atomic charges of water molecule W788.

Residue Asn680 is located at the out edge of the AHBN, connected by two water molecules W786 and W788 through hydrogen bonds, as shown in Fig 4(A). Asn680 has no directive influence on the active residues (Asp511, Glu540, and Asp625), however it may affect the bioactivity of pullulanase through the AHBN. In the site-directed mutation experiments at the position 680, residue Asn was replaced by Asp. Based on the energy minimization calculation, mutation Asn680Asp turns the orientation of water molecule W788, shown in Fig 4(B) and 4(C), and changes the atomic charges of Asn680 (and Asp680), and two water molecules (W786 and W788), listed in Table 2. The changes in orientation and atomic charges may affect the transportation of water molecules and protons to the active center in certain degree.

**Table 2:** Atomic charge changes associating with the mutation Asn680Asp.

	Amide		W786			W788		
Asn680	O	N	O	H_1_	H_2_	O	H_1_	H_2_
	-0.420	-0.782	-0.801	0.410	0.367	-0.750	0.395	0.369
	Carboxyl		W786			W788		
Asp680	O_1_	O_2_	O	H_1_	H_2_	O	H_1_	H_2_
	-.495	-0.579	-0.787	0.406	0.57	-0.783	0.347	0.395

**Fig 5:**
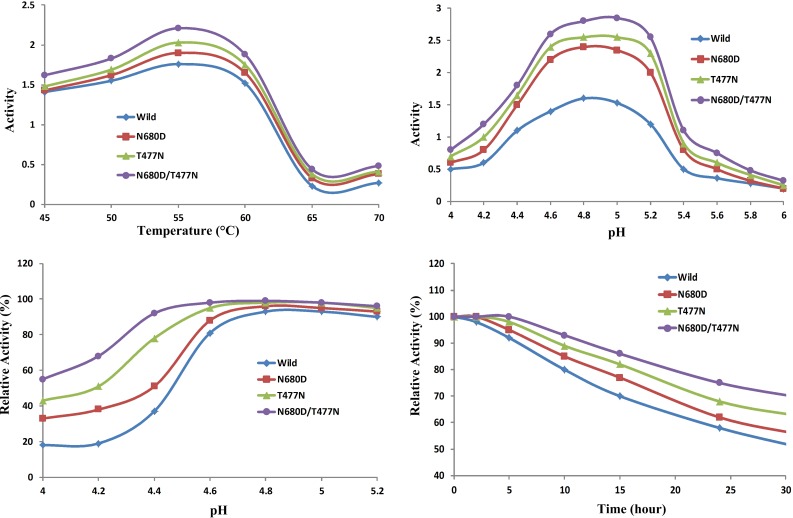
The effects of mutation N680D, T477N, and combined mutation N680D/T477N on the bioactivity of pllulannase BNPulA324. (a) The effect of temperature to the bioactivity of wild type pullulananse and mutated type pullulananse. (b) The effect of pH to the bioactivity of wild type pullulananse and mutated type pullulananse. (c) The stability of bioactivity of pullulanases for pH values. (d) The effect of temperature to the bioactivity of the two pullulanases.

The effects of mutation Asn680Asp on the bioactivity of pullulanase BNPulA324 are shown in Fig 5. In Fig 5(A) the thermostability of mutated pullulanase is little better than the wild type. The temperature range for best bioactivity of mutated type is 50 to 60°C. The effect of pH to the bioactivity of wild type and mutated type are shown in Fig 5(B). The pH range for best bioactivity of mutated type is 4.5 to 5.2, better than that of wild type. Fig 5(C) shows the sensitivity of bioactivity of pullulanases to the pH value. The Asn680Asp mutated type is also better than the wild type. Fig 5(D) shows the bioactive stability of the two pullulanases to the working times. The bioactivity of the mutated type lasts longer time than that of the wild type.

### Effects of Thr477-mutations on enzyme activity

**Fig 6:**
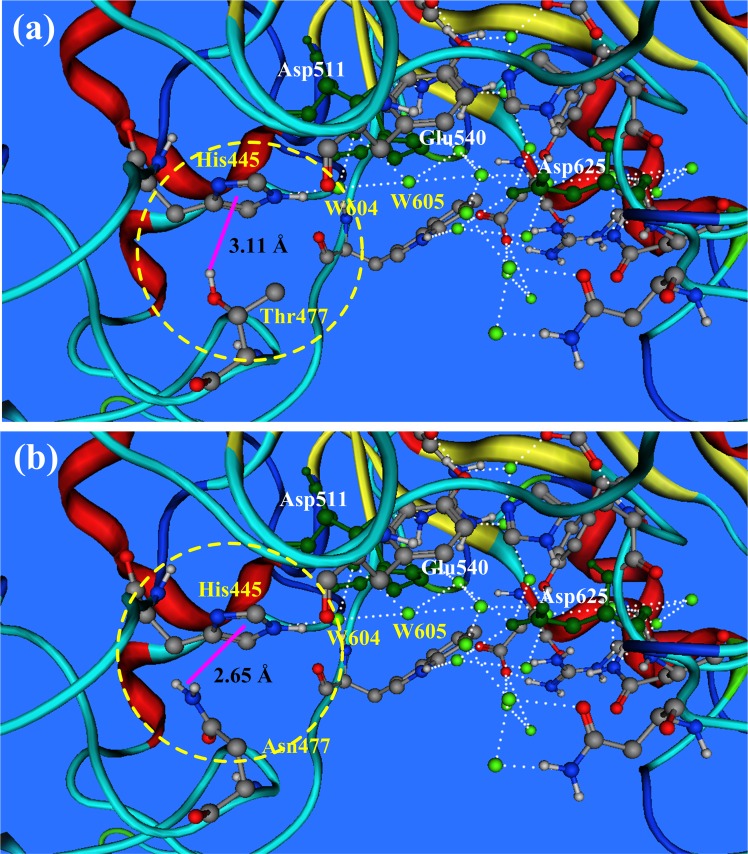
The polar hydrogen-π bond (Hp-π) between His445 and Thr477, and between His445 and Asn477. (a) The hydroxyl group of Thr477 and the imidazole ring of His445 form a stable polar hydrogen-π bond (3.11 Å), indicated by the yellow circle and the pink line. The His445 forms a hydrogen bond with water molecule W604, by which Thr477 and His445 join in the AHBN of pullulanase BNPulA324. (b) When the Thr477 is replaced by Asn477, the amino group (-NH_2_) of Asn477 and the imidazole ring of His445 form a polar hydrogen-π bond (2.65 Å). The catalytic activity of pullulanase BNPulA324 is improved by the mutation Thr477Asn greatly.

The residue Thr477 is located at the out edge of the active hydrogen bond network. The hydroxyl group of Thr477 and the imidazole ring of His445 form a stable polar hydrogen-π bond, even stronger than the common hydrogen bonds [28,34]. On the other hand the His445 forms a hydrogen bond with water molecule W604, shown in Fig 6(A), by which Thr477 and His445 join in the AHBN of pullulanase BNPulA324.

Histidine is the only amino acid playing the roles of both proton donor and acceptor in proteins. In the hydrolysis reaction of pullulanase BNPulA324, His445 could play the role of proton deliver that transports the proton from outside to inside of AHBN. The saturated mutations on Thr477 proved the importance of the residue at the position 477. When the Thr477 is replaced by Asn477, the catalysis activity and pH-stability of pullulanase BNPulA324 are improved greatly.

The polar hydrogen-π bonds between His445 and Thr477 and between His445 and Asn477 are shown in Fig 6(A) and 6(B), indicated by pink lines and yellow circles. After the mutation Thr477Asn, in the hydrogen-π bond the polar hydrogen group is changed from the hydroxyl group (-OH) of Thr477 to the amino group (-NH_2_) of Asn477, and the distance decreases from 3.11 Å to 2.65 Å. The pK_a_ value of His445 is affected by the polar hydrogen-π bond [28,34]. The stronger polar hydrogen-π bond between His445 and Asn477 is favorable to the hydrolytic catalysis reaction of pullulanase BNPulA324.

### Combined mutations of Asn680Asp and Thr477Asn

Both the single point mutations Asn680Asp and Thr477Asn show positive effects for the hydrolysis reaction of pullulanase BNPulA324. A combined mutation of Asn680Asp and Thr477Asn is performed in this section, and the results are shown in Fig 5. The thermal stability, pH-sensitivity, and catalysis activity of pullulanase BNPulA324 are further improved by the combined mutation of Asn680Asp and Thr477Asn.

## Discussion

The relationship between protein structure and its bioactivity is one of the fundamental problems in biological science. For rational protein design and protein engineering we want to know how the changes on protein structures affect its bioactivities. In the catalytic hydrolysis reactions of starch and amylopectin by α-amylases and pullulanases, water molecules and protons are necessary reactants, which have to be transported to the active reaction center of the enzyme along the linkage of hydrogen bond network. Based on above understanding, the active hydrogen bond network (AHBN) method is conceived for protein engineering. AHBN is a three dimensional network covering the active center of an enzyme, connecting the active residues, functional residues, and conservative water molecules with hydrogen bonds.

Even though in many cases the exact transportation mechanism of protons and water molecules is not clear, the efficiency of the catalyst pullulanases to the hydrolytic reactions must be affected by the functional residues in AHBN. Therefore AHBN may help us to find the target residues for site-directed mutations. In the selection of target residues for site-directed mutations the functional residues, located at the out edges of AHBN, (e.g., Thr477 and Asn680), are good candidates, which may pass water molecules and protons from outside to the active region of enzyme. Some of the functional residues (e.g., His445 and His624) in AHBN are very conservative and irreplaceable. In such cases the surrounding residues, which may have influence to the conservative functional residues in AHBN, may be selected as the mutation targets. On the other hand, the functional residues inside the AHBN, such as Arg509 and Glu326, usually are very conservative and important, therefore mutations at these positions may expropriate the bioactivity of enzyme completely.

For catalytic hydrolysis reactions, besides the transportation of water and protons, which is carried out by AHBN, there are other influence factors, for example, the binding interaction between substrate and enzyme, and the transportation of products. For better results of protein engineering, we need combine different tools. The AHBN method, proposed in this study, may provide an assistant and alternative tool for this purpose.

## Supporting Information

S1 File Homology Model of BNPulA324.pdb. This file contains two structures. The first one is the homology model structure of pullulanase BNPulA324 from Bacillus naganoensis. The homology model of pullulanase BNPulA324 was built based on the template pullulanase 2WAN. The second one is the structure of water molecules in protein crystal of BNPulA324 pullulanase. The water molecules were taken from the template structure of pullulanase 2WAN. The orientations of water molecules were adjusted by means of energy minimization calculations.
(PDB)

S2 File BNPulA324-model and 2WAN-Template.pdb. This file contains three structures. The first one is the homology model structure of pullulanase BNPulA324 from Bacillus naganoensis. The second is the experimental structure of template pullulanase 2WAN. The third is the structure of water molecules, whose orientations were adjusted by means of energy minimization calculations based on the homology structure of pullulanase BNPulA324.
(PDB)
